# Proadrenomedullin and copeptin in pediatric pneumonia: a prospective diagnostic accuracy study

**DOI:** 10.1186/s12879-015-1095-5

**Published:** 2015-08-19

**Authors:** Gabriel Alcoba, Sergio Manzano, Laurence Lacroix, Annick Galetto-Lacour, Alain Gervaix

**Affiliations:** Pediatric Emergency Division, Geneva University Hospitals (HUG), Rue Gabrielle Perret-Gentil 6, 1211 Geneva 14, Switzerland; Tropical & Humanitarian Medicine Division, Geneva University Hospitals (HUG), Rue Gabrielle Perret-Gentil 6, 1211 Geneva 14, Switzerland

## Abstract

**Background:**

Community-acquired-pneumonia is the leading cause of child mortality worldwide. Very few studies have explored the predictive value of Proadrenomedullin and Copeptin in pediatric severe pneumonia and bacteremia.

**Methods:**

Proadrenomedullin and Copeptin were assessed as predictors for complicated community-acquired pneumonia (bacteremia, empyema) in 88 children aged 0 to 16 years presenting to the pediatric emergency department, using B.R.A.H.M.S. Kryptor Compact pro-ADM and Copeptin with the TRACE technology (time-resolved amplified cryptase emission). STARD standard reporting was used.

**Results:**

A complicated community-acquired pneumonia was found in 11 out of 88 children (12.5 %). Proadrenomedullin median values increased more than twofold, in complicated vs. uncomplicated (0.18 vs. 0.08 nmol/L, *p* = 0.039), and fivefold in bacteremic vs. non-bacteremic pneumonia (0.40 vs. 0.08 nmol/L, *p* = 0.02). Proadrenomedullin > 0.16 nmol/L showed 100 % sensitivity (95 % CI 39.8 – 100.0) and 70 % (95 % CI 58.7 – 79.7) specificity for bacteremia. Copeptin showed no added-value.

**Conclusions:**

Proadrenomedullin seems a reliable and available predictor for complicated CAP, and could therefore help the physician with the decision to hospitalize, and choose the antibiotics administration route. Larger studies are needed.

**Electronic supplementary material:**

The online version of this article (doi:10.1186/s12879-015-1095-5) contains supplementary material, which is available to authorized users.

## Background

Community-acquired-pneumonia (CAP) is the leading cause of child mortality worldwide [1]. The decision to treat with intravenous antibiotics and to hospitalize will depend on the severity of the disease and the risk of complications such as sepsis, empyema or abscess. Proadrenomedullin (ProADM) and copeptin (CoPEP) are peptides co-synthesized together with adrenomedullin and vasopressin in endothelial cells and pituitary gland respectively. These peptides have vaso-active, immune modulating, and metabolic properties. They are increased in sepsis, but they have a short half-life. ProADM and CoPEP are more stable and easier to measure than the active hormones [2, 3].

**Fig. 1:**
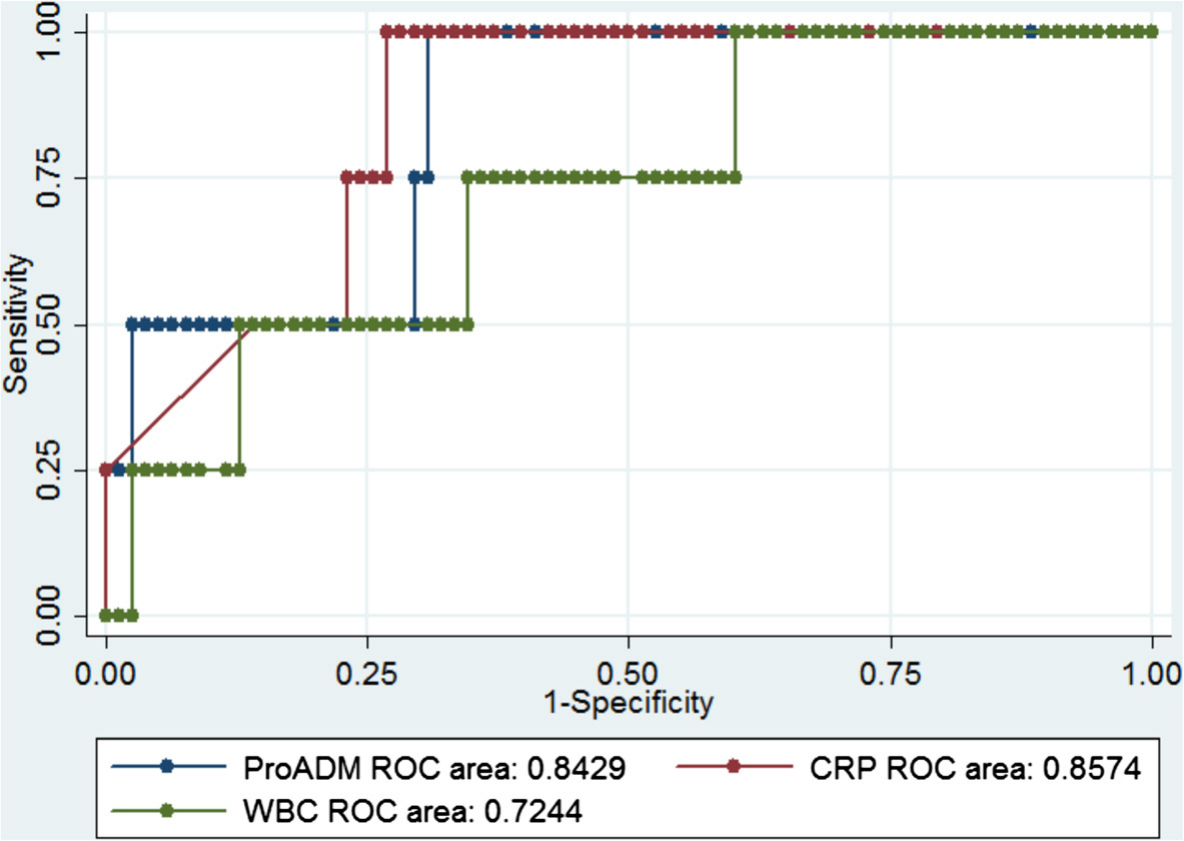
Performance of proadrenomedullin, C-reactive protein, and white blood cell count, for predicting bacteremic pneumonia. ROC area: Receiver-Operator Characteristic area under the curve for sensitivity and 1-specificity to predict bacteremic pneumonia; CRP: C-reactive protein; WBC: white blood cell; ProADM: proadrenomedullin

These novel biomarkers seem useful in predicting severity and complications in severe pneumonia in adults [4–6], but very few studies have explored the predictive value of these markers in children [7, 8].

Our objective was to assess ProADM and CoPEP’s diagnostic accuracy for predicting complications in a cohort of children with community-acquired pneumonia (CAP).

## Methods

### Population and setting

We performed a secondary analysis of a prospective cohort study on biomarkers of pediatric pneumonia [9], including children aged 0 to 16 years presenting to the pediatric emergency department of three tertiary hospitals (Geneva, Lausanne, and Sion) with CAP, defined as fever (>38 °C), cough, tachypnea, and a radiographic lung infiltrate. Children with chronic heart, lung, or neurological diseases were excluded. A venous blood sample was drawn from each child for white blood cell count, C-reactive protein, blood culture (one sample), and subsequently from those with a clear-cut diagnosis of complicated or uncomplicated CAP, we also measured ProADM and CoPEP. Every child had a chest X-ray and, if a pleural effusion was present, a pleural liquid sample was drawn and sent for culture. We defined the diagnosis of “complicated CAP” as CAP with bacteremia (positive blood culture) or empyema (positive pleural culture). CAP with simple pleural effusions without empyema, was considered as “uncomplicated CAP”. The three hospitals ethics committees approved this sub-study within the main pneumonia study [9], (Geneva, Vaud, and Valais states’ Human Research Ethics Committees http://www.swissethics.ch/eks_e.html) and participants’ parents or teenage patients provided informed written consent

### Laboratory methods

Serum samples were immediately stored at −80 °C. ProADM and CoPEP values were determined using TRACE (*time-resolved amplified cryptase emission*) technology with the B.R.A.H.M.S. Kryptor Compact pro-ADM and Copeptin® (Brahms, Hennigsdorf, Germany). White blood cell counts (WBC) with differential (Band neutrophil percentage) and C-reactive protein (CRP) had already been performed in all children. The laboratory team who performed ProADM and CoPEP was blinded to the clinical data.

### Statistics

Using STATA 11.0 (Texas, USA) we analyzed baseline demographic and clinical characteristics expressed as percentages for categorical data, and median with interquartile ranges (IQR) for continuous data due to their non-normal distribution. Statistical associations were assessed using a chi-square test or Fisher exact test for categorical data, and a student’s *t*-test or Wilcoxon-Mann–Whitney rank-sum test for continuous data; and multiple logistic regression using odds-ratios (adjusting for age, sex, previous medication and pneumococcal vaccination) was used to identify variables (ProADM, CoPEP, etc.) independently associated with the outcome variable, namely the cases of complicated CAP. Diagnostic performance was expressed through sensitivity, specificity, positive and negative predictive values (PPV and NPV), positive and negative likelihood ratios (LR+ and LR-) with 95 % Confidence Intervals (95 % CI), and receiver-operator characteristic with area under the curve (AUC). STARD, the Standard Reporting for Diagnostic studies was used, and its checklist is provided in Additional file 1.

## Results

We analyzed 88 samples from children meeting the inclusion criteria for CAP. These children were included from January 2008 to September 2010. Among these 88 children with CAP, eleven (12.5 %) presented with complicated CAP, (9 empyema, 4 bacteremia, and 2 with both complications). The pathogens isolated in blood and empyema cultures were all *S.pneumoniae*. The comparison between the two groups (complicated vs. uncomplicated CAP) showed no significant differences for baseline characteristics: age (median 3.1 years, IQR 1.81 – 5.76), sex, daycare attendance, passive tobacco exposure, and pneumococcal vaccination (*p* > 0.05).

Regarding the main objective, children with bacteremic pneumonia (*n* = 4) or empyema (*n* = 9) showed a general statistical association with ProADM (logistic regression *p* = 0.007 and 0.036 respectively), but not with CoPEP (*p* > 0.05). We found a significant difference in ProADM median values in complicated cases: complicated vs. uncomplicated (0.18 vs. 0.08 nmol/L, *p* = 0.039) and bacteremia vs. no-bacteremia (0.40 vs. 0.08 nmol/L, *p* = 0.02), as shown in the boxplot of Additional file 2.

This shows a twofold increase for complicated and a fivefold increase for bacteremic pneumonia. On the contrary, CoPEP did not distinguish complicated from uncomplicated CAP (*p* = 0.95).

The diagnostic accuracy of these biomarkers in predicting complicated CAP was analyzed and compared with classical biomarkers (WBC, band neutrophils, CRP). We found an optimal cut-off at 0.16 nmol/L for ProADM with 100 % sensitivity (95 % CI 39.8 – 100.0) and 70 % (95 % CI 58.7 – 79.7) and 10 pmol/L for CoPEP with a specificity of 67.5 % (95 % CI 56.1–77.6 %), but a low sensitivity 50 % (6.8–93.2 %). Table 1 shows that ProADM with a 0.16 nmol/L cut-off was very efficient to rule out bacteremia (sensitivity and NPV = 100 %, LR- 0.14 [0.01–2.00]), as accurate as CRP (>100 mg/L) and better than band neutrophils (>1.5 G/L) or leukocytosis (>15 G/L). ProADM >0.16 nmo/L, with a specificity of 70 % for bacteremia, was as accurate as band neutrophils, or CRP and better than leukocytosis to rule it in. With an overall ROC AUC of 0.85, the accuracy of ProADM was comparable to CRP for the diagnosis of bacteremia, as shown in Fig. 1. In contrast, CoPEP did not perform as well to exclude or confirm complicated CAP.

**Table 1:** Performance of proadrenomedullin, copeptin, C-reactive protein, white blood cell and band neutrophils (band cells) counts, for predicting “complicated” (with empyema or bacteremia) and “bacteremic“community-acquired pneumonia

Complicated CAP (*n* = 11; 12.5 %) vs. non-complicated CAP (*n* = 77)					
*N* = 88	Proadrenomedullin >0.16 nmol/L	Copeptin >10 pmol/L	CRP >100 mg/L	White blood cells >15G/L	Band cells >1.5G/L
Sensitivity %	72.7 (39.0–94.0)	45.5 (16.7–76.6)	100.0 (71.5–100)	72.7 (39.0–94.0)	90.9 (58.7–99.8)
Specificity %	71.4 (60.0–81.2)	68.8 (57.3–78.9)	77.0 (65.8–86.0)	58.7 (46.7–69.9)	75.7 (64.3–84.9)
LR+	2.55 (1.5–4.2)	1.46 (0.70–3.0)	4.11 (2.68–6.29)	1.76 (1.12–2.76)	3.55 (2.26–5.56)
LR-	0.38 (0.1–1.0)	0.79 (0.45–1.39)	0.05 (0.00–0.82)	0.46 (0.17–1.24)	0.17 (0.04–0.75)
PPV%	26.7 (12.3–45.9)	17.2 (5.8–35.8)	39.3 (21.5–59.4)	20.5 (9.3–36.5)	35.7 (18.6–55.9)
NPV%	94.8 (85.6–98.9)	89.8 (79.2–96.2)	100.0 (93.7–100)	93.6 (82.5–98.7)	98.2 (90.6–100)
ROC area	0.72 (0.6–0.9)	0.57 (0.41–0.73)	0.89 (0.84–0.93)	0.66 (0.51–0.81)	0.83 (0.73–0.93)
Odds ratio^a^	6.67 (1.7–25.3)	1.84 (0.54–6.30)	75.6 (4.2–1348)	3.78 (1.00–14.2)	21.4 (3.57–128)
Bacteremic CAP (*n* = 4; 4.5 %) vs. non-bacteremic CAP (*n* = 84)					
*N* = 88	Proadrenomedullin >0.16 nmol/L	Copeptin >10 pmol/L	CRP >100 mg/L	White blood cells >15G/L	Band cells >1.5G/L
Sensitivity %	100 (39.8–100.0)	50.0 (6.8–93.2)	100 (39.8–100)	75.0 (19.4–99.4)	75.0 (19.4–99.4)
Specificity %	70.0 (58.7–79.7)	67.5 (56.1–77.6)	70.5 (59.1–80.3)	57.0 (45.3–68.1)	71.8 (60.5–81.4)
LR+	2.98 (1.91–4.63)	1.54 (0.55–4.31)	3.03 (1.93–4.73)	1.62 (0.87–3.04)	2.46 (1.26–4.81)
LR-	0.14 (0.01–2.00)	0.74 (0.27–2.00)	0.14 (0.01–1.98)	0.53 (0.14–2.04)	0.42 (0.11–1.61)
PPV %	14.3 (4.0–32.7)	7.1 (0.9–23.5)	14.8 (4.2–33.7)	8.1 (1.7–21.9)	12.0 (2.5–31.2)
NPV %	100.0 (93.6–100.0)	96.4 (87.7–99.6)	100 (93.5–100)	97.8 (88.5–99.9)	98.2 (90.6–100)
ROC area	0.85 (0.80–0.90)	0.59 (0.30–0.88)	0.85 (0.80–0.90)	0.66 (0.41–0.91)	0.73 (0.48–0.98)
Odds ratio^a^	20.8 (1.1–400.5)	2.08 (0.35–12.5)	21.3 (1.1–411)	3.08 (0.43–21.9)	5.86 (0.81–42.2)

*CAP* Community-acquired pneumonia, *CRP* C-reactive protein, *ROC area* Receiver-Operator Characteristic area under the curve, *LR+* and *LR* Likelihood ratio of a positive or negative result, *NPV* and *PPV* Negative and Positive Predictive Values (NPV, PPV); ^a^All odds ratios (OR) are multivariable-adjusted-OR (age, sex, previous medication and pneumococcal vaccination)

## Discussion

This secondary analysis of 88 children with CAP supported the hypothesis that ProADM strongly predicts serious complications of pediatric community-acquired pneumonia (CAP), such as bacteremia and empyema. CoPEP on the contrary did not show such performances for predicting complicated CAP in our small population, but should be retested in larger samples.

Diagnostic performances of ProADM seem excellent, especially to rule-out bacteremia (sensitivity 100 %), and as accurate as classical markers (WBC, Band neutrophils, and CRP) with regards to specificity. Only true complications, such as bacteremia or empyema, seem to cause a significant elevation of ProADM above 0.16 nmol/L.

Compared to similar studies, Renaud et al. [6] showed that ProADM in elderly patients improved the prediction of early admission to ICU for severe CAP. In their study, ProADM showed a sensitivity of 95.0 % at a cut-off value of 0.75 nmol/L, and a specificity of 81.0 % at a cut-off value of 2.0 nmol/L.

One pediatric study by Sardà Sanchez et al. [8] showed that ProADM could predict simple pneumonia versus pneumonia with complications (2.32 vs. 1.18 nmol/L, *p* = 0.014) or pleural effusion (2.94 vs. 1.14, *p* < 0.001), but only fifty patients were included in this study, including ten with complications, of which seven with pleural effusions. Another pediatric study by Michels et al. [7] concluded that ProADM also predicts capillary damage, leakage and risk of shock in hemorrhagic dengue and dengue shock, showing that these lesions similar to sepsis can be predicted by this marker in non-bacterial sepsis-like syndromes.

Initially a marker of severity in non-infectious diseases such as stroke, CoPEP also seemed promising as a prognostic marker in CAP. This was suggested by Katan et al.’s review on adults with ventilator-associated pneumonia [5], and in a Swiss study on lower respiratory tract infections by Müller B et al. [10]. In the latter, CoPEP levels were significantly lower in survivors. However, in our study CoPEP did not show any added value as a predictor for complicated pediatric CAP, but the sample size may limit such conclusions. This could also be due to the low prevalence of severe CAP in our population compared to that of intensive care units in these studies, and the absence of mortality, even in cases with bacteremia and empyema. This hypothesis should be tested in future studies among children with ventilator associated pneumonia or sepsis.

A few limitations need to be stated: the design of this study was a secondary analysis of a larger study focused on the etiology of childhood CAP, with selection of samples with typical characteristics rather than random selection, implying a slight risk of selection bias. Although most statistical analyses seem coherent and logical compared to other studies, the design of this study did not include a sample size calculation, so that the significance levels could be inaccurate in small groups. The laboratory team who performed ProADM and CoPEP was blinded to the clinical data, reducing the risk of bias.

## Conclusion

In conclusion, we found that ProADM, in contrast to CoPEP, seems to be an interesting marker of complicated CAP, very similar to CRP in sensitivity and specificity. It could therefore help the physician with the decision to hospitalize and choose the antibiotics administration route. Larger studies should assess the promising performances of ProADM in pneumonia and other serious pediatric infections, as well as further comparisons with CRP and Procalcitonin.

## Additional files

Additional file 1:
**STARDchecklist_wordversion.** (DOC 48 kb) Additional file 2:
**Box plot.** (PPTX 56 kb)
