# Supplementary figures and images for: Proadrenomedullin and copeptin in pediatric pneumonia: a prospective diagnostic accuracy study
Source: BMC Infect Dis. 2015 Aug 19;15:347. doi: 10.1186/s12879-015-1095-5 (PMC4543464; doi:10.1186/s12879-015-1095-5)

## Slide 1
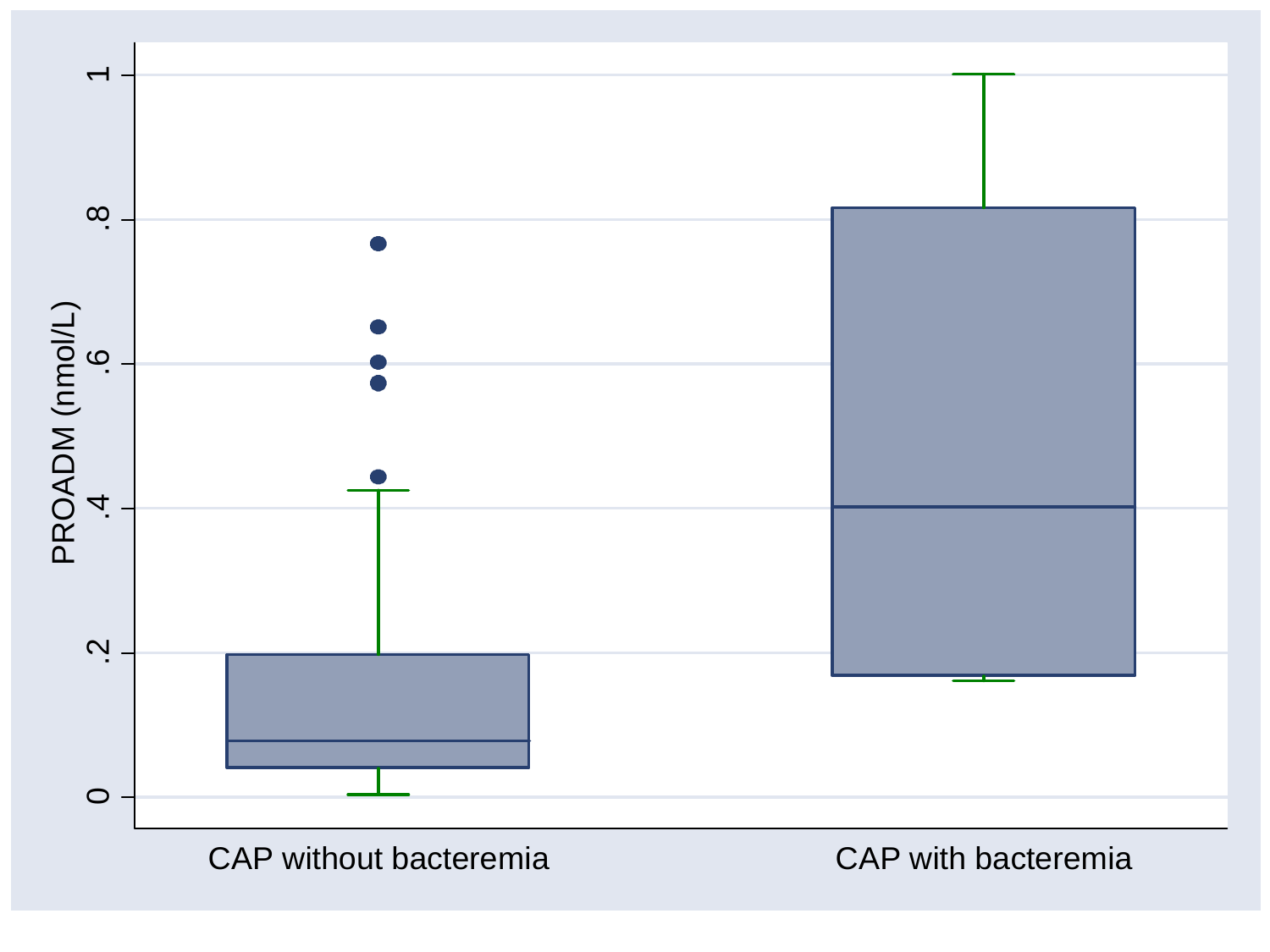

Supplement: Additional file 2: — Box plot. (PPTX 56 kb) [file 12879_2015_1095_MOESM2_ESM.pptx]
